# Systemic gene therapy corrects the neurological phenotype in a mouse model of NGLY1 deficiency

**DOI:** 10.1172/jci.insight.183189

**Published:** 2024-10-08

**Authors:** Ailing Du, Kun Yang, Xuntao Zhou, Lingzhi Ren, Nan Liu, Chen Zhou, Jialing Liang, Nan Yan, Guangping Gao, Dan Wang

**Affiliations:** 1 Horae Gene Therapy Center, University of Massachusetts Chan Medical School, Worcester, Massachusetts, USA.; 2 Department of Immunology and; 3 Department of Microbiology, University of Texas Southwestern Medical Center, Dallas, Texas, USA.; 4 Department of Microbiology and Physiological Systems and; 5 RNA Therapeutics Institute, University of Massachusetts Chan Medical School, Worcester, Massachusetts, USA.

**Keywords:** Neuroscience, Therapeutics, Gene therapy, Neurodegeneration, Neurological disorders

## Abstract

The cytoplasmic peptide:N-glycanase (NGLY1) is ubiquitously expressed and functions as a de–N-glycosylating enzyme that degrades misfolded N-glycosylated proteins. NGLY1 deficiency due to biallelic loss-of-function *NGLY1* variants is an ultrarare autosomal recessive deglycosylation disorder with multisystemic involvement; the neurological manifestations represent the main disease burden. Currently, there is no treatment for this disease. To develop a gene therapy, we first characterized a tamoxifen-inducible *Ngly1*-knockout (*iNgly1*) C57BL/6J mouse model, which exhibited symptoms recapitulating human disease, including elevation of the biomarker GlcNAc-Asn, motor deficits, kyphosis, Purkinje cell loss, and gait abnormalities. We packaged a codon-optimized human *NGLY1* transgene cassette into 2 adeno-associated virus (AAV) capsids, AAV9 and AAV.PHPeB. Systemic administration of the AAV.PHPeB vector to symptomatic *iNgly1* mice corrected multiple disease features at 8 weeks after treatment. Furthermore, another cohort of AAV.PHPeB-treated *iNgly1* mice were monitored over a year and showed near-complete normalization of the neurological aspects of the disease phenotype, demonstrating the durability of gene therapy. Our data suggested that brain-directed *NGLY1* gene replacement via systemic delivery is a promising therapeutic strategy for NGLY1 deficiency. Although the superior CNS tropism of AAV.PHPeB vector does not translate to primates, emerging AAV capsids with enhanced primate CNS tropism will enable future translational studies.

## Abstract 2

Systemic AAV.PHPeB administration improved neurological symptoms in NGLY1-deficient mice, showing promise for treating this rare disorder.

## Introduction

**Figure 1:**
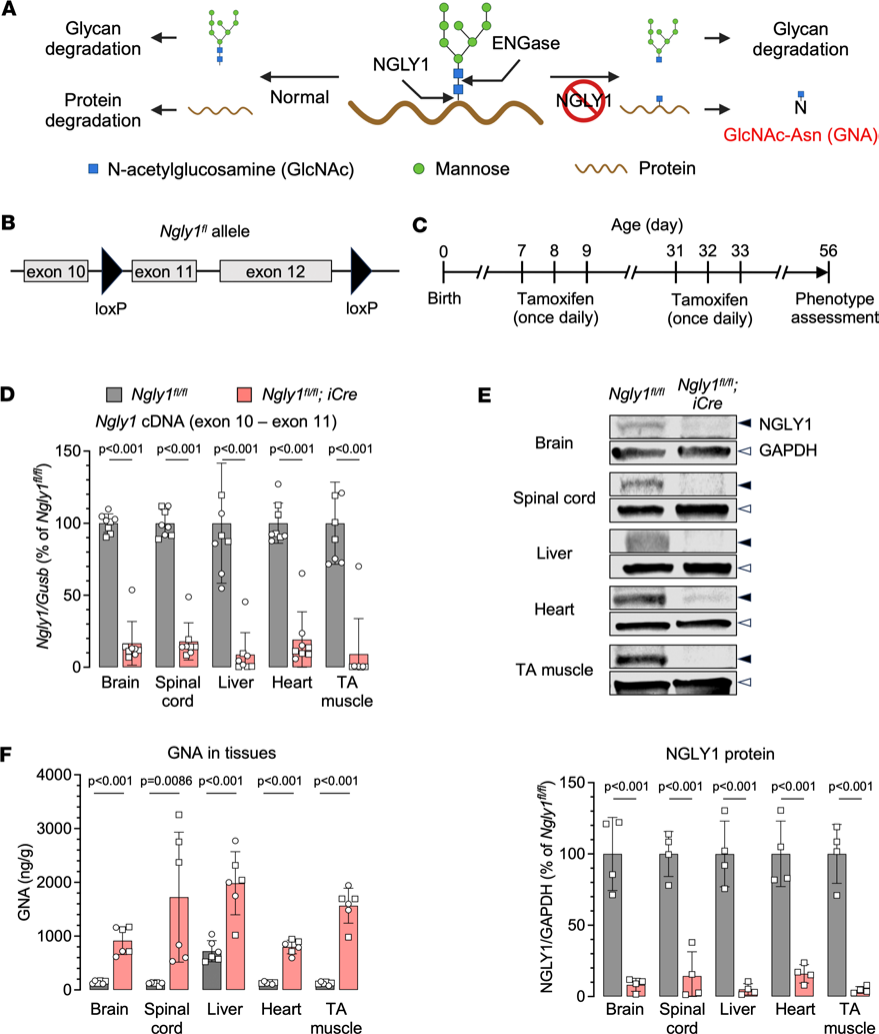
NGLY1 knockdown in an inducible knockout mouse model of NGLY1 deficiency. (**A**) Schematic cartoon showing the biological function of NGLY1 and the generation of N-GlcNAc (GNA) in its absence. ENGase, endo-β-N-acetylglucosaminidase. (**B**) A part of the genome structure of mouse *loxP*-flanked (flox or fl) *Ngly1^fl^* allele. The endogenous *Ngly1* locus carries 2 *loxP* sites flanking exons 11 and 12. (**C**) Timeline of tamoxifen administration and workflow to induce *Ngly1* conditional knockout in mice. (**D**) Quantification of endogenous mouse *Ngly1* mRNA expression (cDNA) in the brain, spinal cord, liver, heart, and tibialis anterior (TA) muscle of *Ngly1^fl/fl^* and *Ngly1^fl/fl^ iCre* mice. All mice were treated with tamoxifen as shown in **C** and euthanized on postnatal day 56 (P56). *N* = 8 mice (4 males and 4 females) per group. (**E**) Representative Western blotting images of NGLY1 protein expression in the brain, spinal cord, liver, heart, and TA muscle of the mice as shown in **D** (males only). Quantification of NGLY1 signal (normalized to GAPDH expression) is shown below the images. *N* = 4 mice per group. (**F**) GNA levels in various tissues of *Ngly1^fl/fl^* mice and *Ngly1^fl/fl^ iCre* mice. *N* = 6 mice (3 males and 3 females) per group. In **D**–**F** data are mean ± SD of biological repeats; each white dot represents an individual mouse (circle: female; square: male). Statistical analysis was performed by 2-tailed Student’s *t* test.

N-glycanase 1 (NGLY1) deficiency (OMIM #615273) is an ultrarare autosomal recessive deglycosylation disorder with multisystemic involvement, including global developmental delay, movement disorder, a reduction or lack of tears, transient elevation of liver enzymes, and scoliosis (1–9). More than 100 patients have been reported worldwide. It is caused by biallelic loss-of-function variants in the *NGLY1* gene (10–13) that encodes peptide:N-glycanase (PNGase, aka N-glycanase 1) (14–16). NGLY1 plays an integral role in the endoplasmic reticulum–associated degradation pathway by clearing misfolded glycoproteins. It cleaves the aspartyl glycosylamine bond of N-linked glycoproteins to produce a free oligosaccharide and a deaminated protein, which are further degraded in the lysosome and by the proteasome, respectively (17–19) (Figure 1A). Under NGLY1 deficiency, another deglycosylating enzyme known as ENGase removes N-glycans by cleaving the glycosidic bond between the 2 GlcNAc residues, leaving a single GlcNAc residue attached to the protein. This incompletely deglycosylated protein is prone to aggregation (17, 20), contributing to dysregulation of several cellular processes and pathogenesis. Furthermore, degradation of GlcNAc-protein generates GNA that is inversely correlated to the NGLY1 function (17, 21) (Figure 1A). GNA levels are consistently elevated in NGLY1-deficient cells, animal models, and patients and can serve as a biomarker for NGLY1 deficiency (22–24).

The pathogenesis of NGLY1 deficiency is still poorly understood, and no effective therapy is currently available. Intriguingly, *ENGase* gene deletion can reverse abnormal GlcNAc-protein accumulation in *Ngly1*-knockout mouse embryonic fibroblasts (25) and partially rescue the lethality of NGLY1-deficient mice (26). Several small molecule ENGase inhibitors were discovered in the FDA-approved drug database and considered as potential treatments for NGLY1 deficiency (27). Besides clearing misfolded proteins, recent reports showed that the deglycosylation function of NGLY1 also regulates biologically relevant proteins, such as nuclear factor erythroid-2-like 1 (28–32) and Na^+^-K^+^-Cl^−^ co-transporter 1 (33, 34), and cellular pathways, such as BMP signaling (35, 36) and AMPK signaling (37, 38). In addition, the nonenzymatic, regulatory function of NGLY1 in aquaporin transcription has been reported (39). The pathophysiological basis of NGLY1 deficiency may be associated with the absence of various enzymatic and nonenzymatic roles of NGLY1 (40). Therefore, gene replacement therapy may achieve maximal NGLY1 function restoration by addressing the root cause of NGLY1 deficiency.

In a previous study, Asahina et al. generated recombinant adeno-associated virus serotype 9 (rAAV9) vector expressing human *NGLY1* cDNA and tested its therapeutic efficacy in an *Ngly1*-knockout (*Ngly1^–/–^*) rat model. A single intracerebroventricular (ICV) administration at the dose of 2 × 10^10^ vector genomes (vg)/rat restored NGLY1 expression in the brain and spinal cord and normalized the motor phenotype of *Ngly1*^–*/*–^ rats (41, 42). More recently, Zhu et al. compared 3 routes of administration of an AAV9-based gene therapy vector (GS-100) in the same rat model, namely intravenous (IV), ICV, and IV+ICV, and found that ICV and IV+ICV administration resulted in widespread transgene delivery and expression throughout the CNS, concomitant with significant reduction in GNA in the CNS and behavioral improvements compared with untreated *Ngly1^–/–^* rats. IV-only administration did not provide any benefit, and IV+ICV did not provide additional benefit compared with ICV only (43). Together, these studies suggest that CNS-directed gene therapy is a promising treatment strategy for NGLY1 deficiency.

Although ICV administration of rAAV9 leads to widespread transgene delivery throughout rodent brains, achieving the same targeting efficiency in the human brain can be challenging because of its much larger size (44). Unlike certain lysosomal enzymes that can be secreted and taken up by other cells, the cytosolic NGLY1 protein functions in a cell-autonomous manner. Therefore, *NGLY1* gene therapy only rescues transduced cells, and a broader coverage of CNS gene delivery likely correlates with a better therapeutic outcome. Consistent with this notion, ICV delivery of GS-100 at a very high dose of 6 **×** 10^12^ vg/rat only resulted in 50% or less GNA reduction in CNS tissues of *Ngly1^–/–^* rats, presumably limited by the number of transduced cells (43). Systemic delivery of certain rAAVs that can cross the blood-brain barrier (BBB) has the potential to broadly transduce the CNS owing to its high density of capillaries. However, IV delivery of GS-100 at a high dose of 1 × 10^14^ vg/kg to *Ngly1^–/–^* rats did not result in sufficient CNS transduction to confer any therapeutic benefit (43). Recently, several groups engineered AAV capsids to cross the BBB more efficiently than AAV9 in mice (45–47) or nonhuman primates (NHPs) (48–51), potentially enabling noninvasive systemic gene therapy development for myriad neurological disorders afflicting broad CNS regions, such as NGLY1 deficiency.

In this study, we set out to test whether systemic delivery of a potent BBB-crossing rAAV can lead to therapeutic outcome in an NGLY1 deficiency animal model. To this end, we turned to the well-characterized AAV.PHPeB capsid that exhibits 50- to 100-fold higher CNS transduction than AAV9 following systemic injection in C57BL/6 mice (46). Because the superior BBB-crossing property of AAV.PHPeB does not translate to rats (52–54), and *Ngly1^–/–^* mice on C57BL/6 background are embryonically lethal (26), we first generated an inducible *Ngly1*-knockout C57BL/6 mouse model (*iNgly1*). In the *iNgly1* mouse model, the last 2 exons of *Ngly1* are flanked by a pair of *loxP* sites (*Ngly1^fl/fl^*) (17, 30, 55) (Figure 1B), and it carries a tamoxifen-inducible, ubiquitously expressed *Cre* transgene (*iCre*). Therefore, tamoxifen treatment leads to whole-body *Ngly1* knockout. We found that *iNgly1* mice exhibited a range of disease-related phenotypes following tamoxifen treatment, including movement disorder, neurological impairment, scoliosis, and reduced fitness and survival. Systemic injection of an AAV.PHPeB gene replacement vector after disease onset significantly ameliorated the disease phenotype up to 14 months of age. Our data suggested that CNS-directed *NGLY1* gene replacement therapy via systemic delivery is a promising therapeutic strategy for NGLY1 deficiency.

## Results

### Ngly1 knockdown in iNgly1 mice led to a disease-related phenotype.

**Figure 2:**
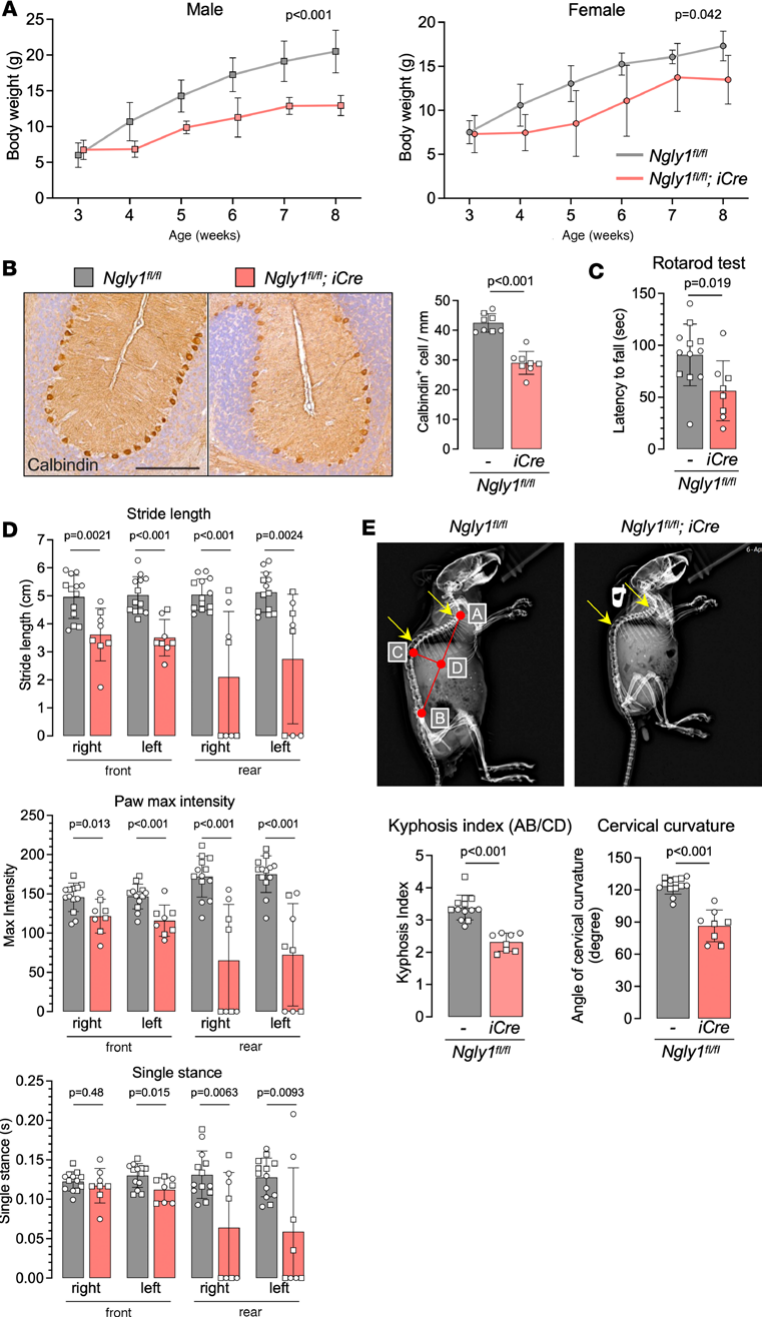
NGLY1 knockdown in *iNgly1* mice leads to disease-related phenotype. (**A**) Body weight of *Ngly1^fl/fl^* and *Ngly1^fl/fl^ iCre* mice as shown in Figure 1C. Data are reported as the mean ± SD of 4–9 mice per group. (**B**) Representative immunohistochemistry (IHC) images of Calbindin, a cellular marker for Purkinje cells, in the cerebellum of *Ngly1^fl/fl^* and *Ngly1^fl/fl^ iCre* mice. Quantification of Calbindin^+^ Purkinje cells is shown to the right of the images. *N* = 8 mice (4 males and 4 females) per group. Scale bar: 200 μm. (**C**) Rotarod test for *Ngly1^fl/fl^* and *Ngly1^fl/fl^ iCre* mice. *N* = 8–12 mice (4 to 5 males and 4 to 7 females) per group. (**D**) Quantification of stride length, paw max intensity, and paw single stance in the CatWalk gait analysis for *Ngly1^fl/fl^* and *Ngly1^fl/fl^ iCre* mice. *N* = 8 to 13 mice (4 to 7 males and 4 to 6 females) per group. (**E**) Representative x-ray images showing kyphosis (yellow arrows). Quantification of kyphosis index (KI; the ratio of AB to CD) and cervical curvature angle is shown below the images. *N* = 8 to 13 mice (4 to 7 males and 4 to 6 females) per group. In **A**–**E**, data are mean ± SD of biological repeats; each white dot represents an individual mouse (circle: female; square: male). In **A**, statistical analysis was performed by 2-way ANOVA, followed by Tukey’s multiple comparisons test. In **B**–**E**, statistical analysis was performed by 2-tailed Student’s *t* test.

To induce *Ngly1* knockout, *iNgly1* mice received 2 rounds of tamoxifen treatment during postnatal day 7 (P7) to P9 and P31 to P33, respectively; their phenotype was assessed on P56 (Figure 1C). Quantification of *Ngly1* mRNA and NGLY1 protein in multiple tissues demonstrated efficient gene expression knockdown by 80%–90% in the *iNgly1* mice compared with the *Ngly1^fl/fl^* littermates (i.e., without the *iCre* transgene and treated with tamoxifen in the same manner) (Figure 1, D and E). Correspondingly, elevated GNA levels were observed in all tissues examined, including brain, spinal cord, liver, heart, and TA muscle (Figure 1F). Compared with *Ngly1^fl/fl^* controls, the *iNgly1* mice showed lower body weight, reduced number of Calbindin^+^ Purkinje cells in the cerebellum, and motor function abnormalities as revealed by rotarod test and CatWalk gait analysis (Figure 2, A–D). They also exhibited severe kyphosis as revealed by x-ray radiography (Figure 2E). Together, these results demonstrated that the *iNgly1* mouse model recapitulated the key clinical features of NGLY1 deficiency in human patients, such as motor impairment and scoliosis.

### Short-term assessment on therapeutic efficacy of systemic gene therapy.

**Figure 3:**
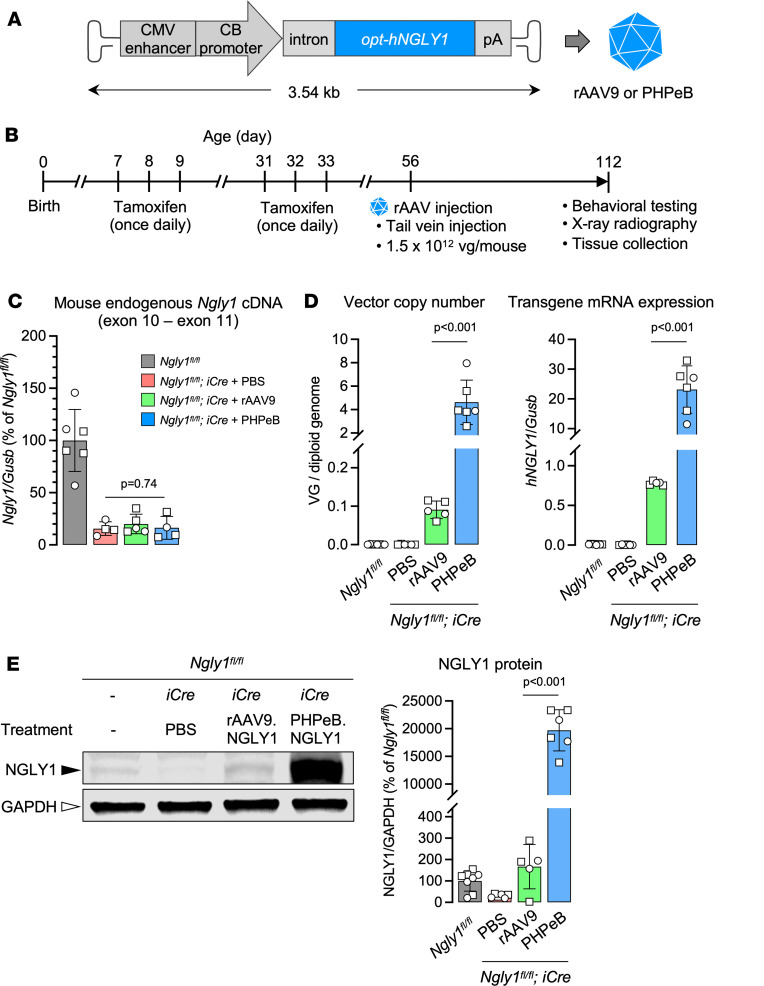
Comparing rAAV9 and rAAV.PHPeB for gene delivery and expression efficiency. (**A**) Schematic diagram showing the vector constructs expressing the codon-optimized human *NGLY1* gene (*opt-hNGLY1*). (**B**) Timeline of tamoxifen administration and workflow to induce *Ngly1* knockout in mice followed by rAAV treatment and phenotype assessment. (**C**) Quantification of mouse endogenous *Ngly1* mRNA expression (cDNA) in the brain. *N* = 4–6 mice per group. (**D**) Quantification of vector genome copy and *hNGLY*1 transcript (reverse-transcribed into cDNA) in the brain. (**E**) Representative Western blotting images of NGLY1 protein expression in the brain of the mice as shown in **C**. Quantification of NGLY1 signal (normalized to GAPDH expression) is shown. *N* = 5 to 8 mice per group. In **C**–**E**, data are mean ± SD of biological repeats; each white dot represents an individual mouse (circle: female; square: male). Statistical analysis was performed by 1-way ANOVA followed by Tukey’s multiple comparisons test.

We designed a gene replacement vector construct that expresses codon-optimized human *NGLY1* cDNA driven by a ubiquitous promoter (*opt-hNGLY1*) (Figure 3A) and packaged it in AAV9 and PHPeB, an engineered capsid showing more efficient CNS transduction than AAV9 by IV injection in C57BL/6 mice (46). Following the same tamoxifen treatment regimen to induce endogenous *Ngly1* knockout, rAAV was injected to mice via tail vein at 1.5 × 10^12^ vg/mouse on P56 (Figure 3B), when *iNgly1* mice showed a disease-related phenotype (Figure 2). Eight weeks later (i.e., on P112), mice were subjected to molecular analysis and phenotype assessment. Consistent with the phenotype characterization study (Figure 1D), the endogenous *Ngly1* mRNA in the *iNgly1* brain was reduced by 80%–90% regardless of rAAV treatment (Figure 3C). As expected, rAAV.PHPeB treatment led to approximately 40-fold higher vector genome copy numbers and *opt-hNGLY1* expression in the brain than rAAV9 (Figure 3, D and E).

**Figure 4:**
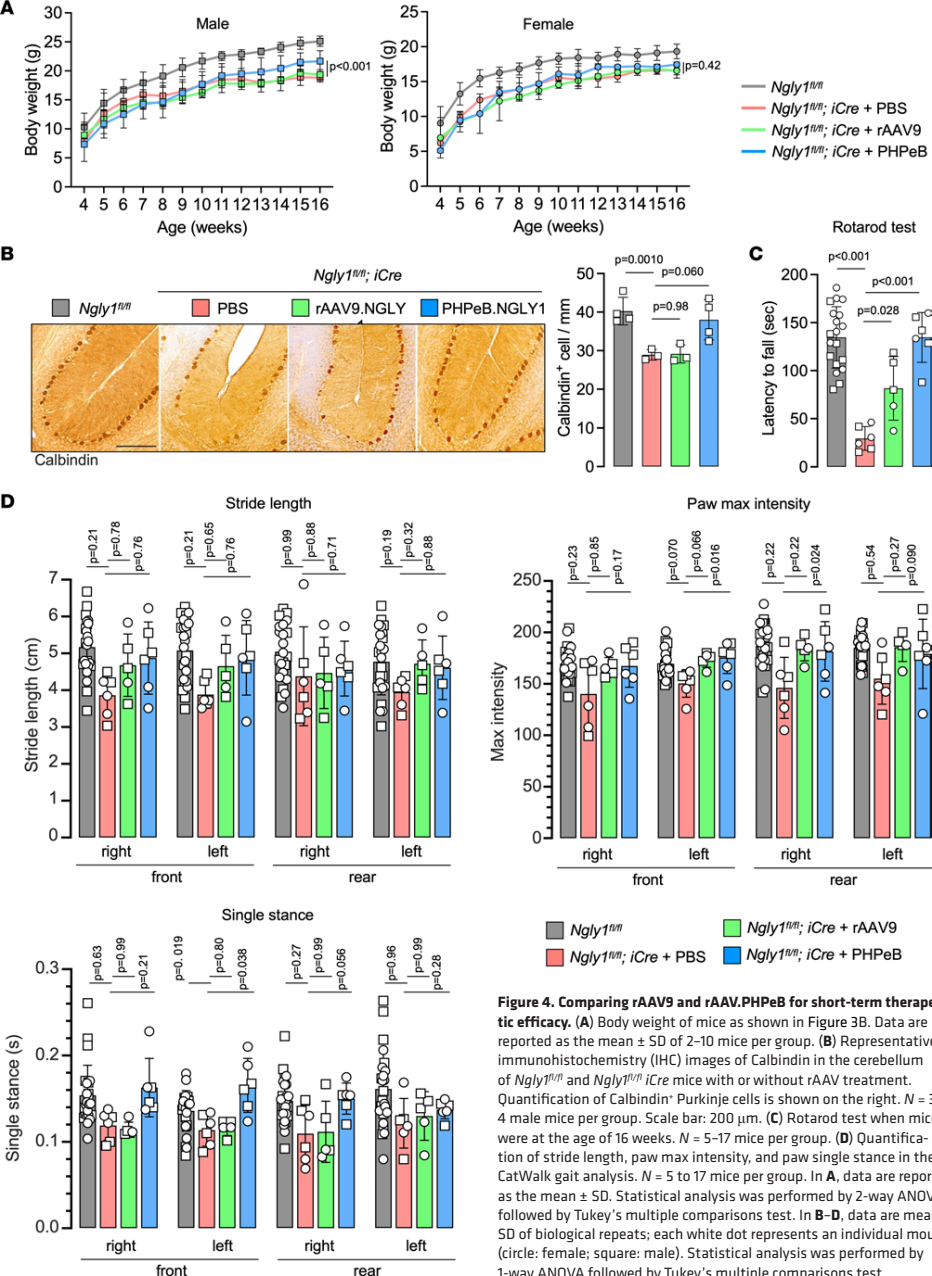
Comparing rAAV9 and rAAV.PHPeB for short-term therapeutic efficacy. (**A**) Body weight of mice as shown in Figure 3B. Data are reported as the mean ± SD of 2–10 mice per group. (**B**) Representative immunohistochemistry (IHC) images of Calbindin in the cerebellum of *Ngly1^fl/fl^* and *Ngly1^fl/fl^ iCre* mice with or without rAAV treatment. Quantification of Calbindin^+^ Purkinje cells is shown on the right. *N* = 3 to 4 male mice per group. Scale bar: 200 μm. (**C**) Rotarod test when mice were at the age of 16 weeks. *N* = 5–17 mice per group. (**D**) Quantification of stride length, paw max intensity, and paw single stance in the CatWalk gait analysis. *N* = 5 to 17 mice per group. In **A**, data are reported as the mean ± SD. Statistical analysis was performed by 2-way ANOVA, followed by Tukey’s multiple comparisons test. In **B**–**D**, data are mean ± SD of biological repeats; each white dot represents an individual mouse (circle: female; square: male). Statistical analysis was performed by 1-way ANOVA followed by Tukey’s multiple comparisons test.

**Figure 5:**
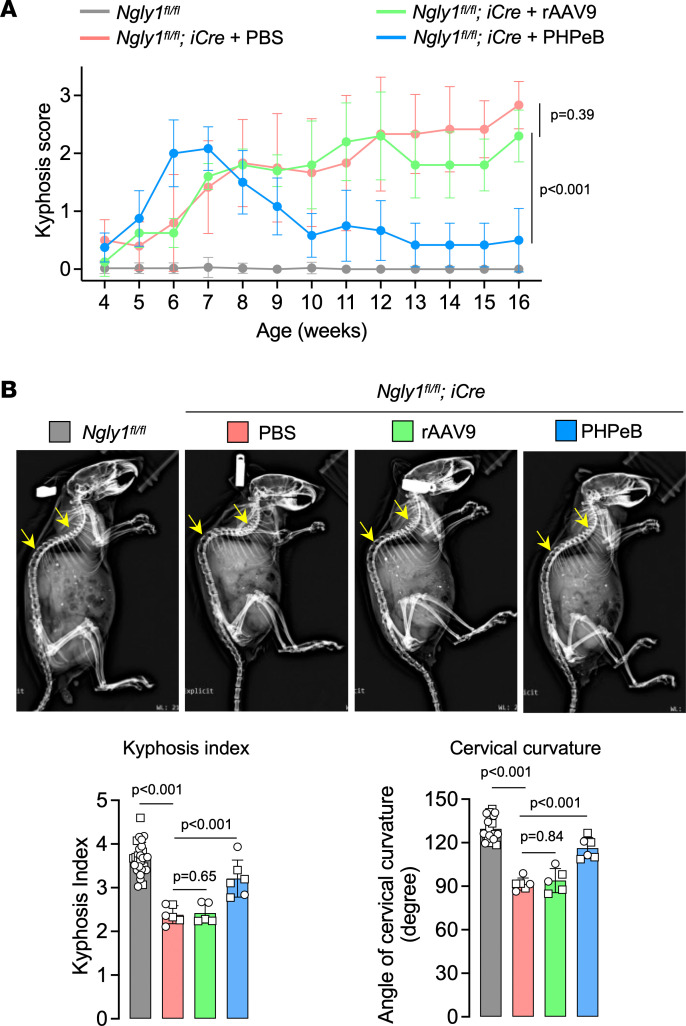
rAAV.PHPeB outperforms rAAV9 in correcting kyphosis. (**A**) Kyphosis scores of *Ngly1^fl/fl^* mice and *Ngly1^fl/fl^ iCre* mice with or without rAAV treatment. *N* = 5–17 mice per group. (**B**) Representative x-ray images showing kyphosis (yellow arrows). Quantification of kyphosis index (KI) and cervical curvature angle is shown below the images. *N* = 5 to 17 mice per group. In **A**, data are reported as the mean ± SD. Statistical analysis was performed by 2-way ANOVA, followed by Tukey’s multiple comparisons test. In **B**, data are mean ± SD of biological repeats; each white dot represents an individual mouse (circle: female; square: male). Statistical analysis was performed by 1-way ANOVA followed by Tukey’s multiple comparisons test.

Although rAAV9 treatment did not improve body weight of *iNgly1* mice, rAAV.PHPeB treatment resulted in marginal body weight gain in males (Figure 4A). The superior therapeutic efficacy of rAAV.PHPeB was evidenced by restoration of Calbindin^+^ Purkinje cells in the cerebellum and normalized performance on rotarod (Figure 4, B and C). In the CatWalk gait analysis, mice receiving rAAV.PHPeB treatment showed equal or better correction compared with rAAV9, though the trend did not reach statistical significance after correcting for multiple comparisons (Figure 4D). Kyphosis was largely rescued by rAAV.PHPeB treatment but not by rAAV9 (Figure 5, A and B). These results led us to conclude that CNS gene delivery was of utmost importance to ameliorate the disease phenotype in *iNgly1* mice. We therefore chose rAAV.PHPeB in the following long-term study.

### rAAV.PHPeB treatment showed long-term therapeutic efficacy in iNgly1 mice.

**Figure 6:**
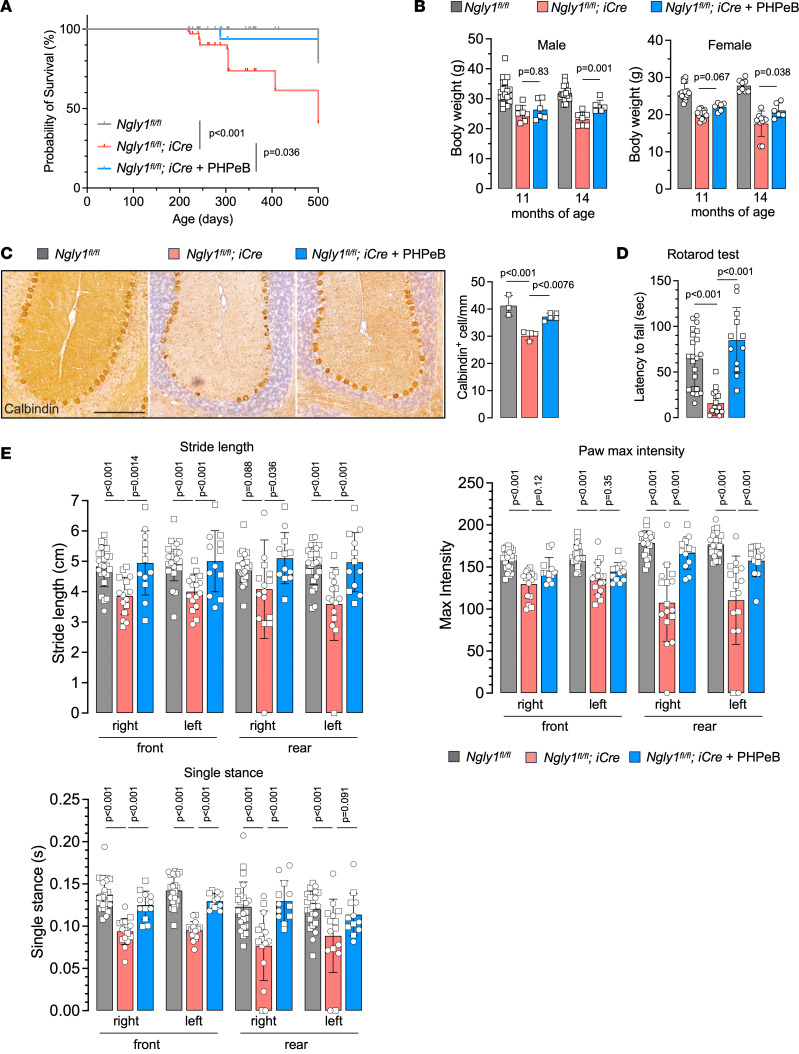
rAAV.PHPeB treatment shows long-term therapeutic efficacy in *iNgly1* mice. (**A**) Kaplan-Meier plot of the survival rate of *Ngly1^fl/fl^* mice and *Ngly1^fl/fl^ iCre* mice with or without rAAV treatment. All mice received tamoxifen treatment as indicated in Figure 3B. *N* ≥ 18 mice per group with similar female/male ratios in each group. (**B**) Body weight at the age of 11 and 14 months. *N* ≥ 6 mice per group. (**C**) Representative immunohistochemistry (IHC) images of Calbindin, a cellular marker for Purkinje cells, in the cerebellum. Quantification of Calbindin^+^ Purkinje cells is shown on the right. *N* = 3 to 5 male mice per group. Scale bar: 200 μm. (**D**) Rotarod test when mice were at the age of 14 months. *N* = 12 to 21 mice per group. (**E**) Quantification of stride length, paw max intensity, and paw single stance in the CatWalk gait analysis. *N* = 12 to 24 mice per group. In **A**, statistical analysis was performed by log-rank (Mantel-Cox) test. In **B**–**E**, data are mean ± SD of biological repeats; each white dot represents an individual mouse (circle: female; square: male). Statistical analysis was performed by 1-way ANOVA followed by Tukey’s multiple comparisons test.

**Figure 7:**
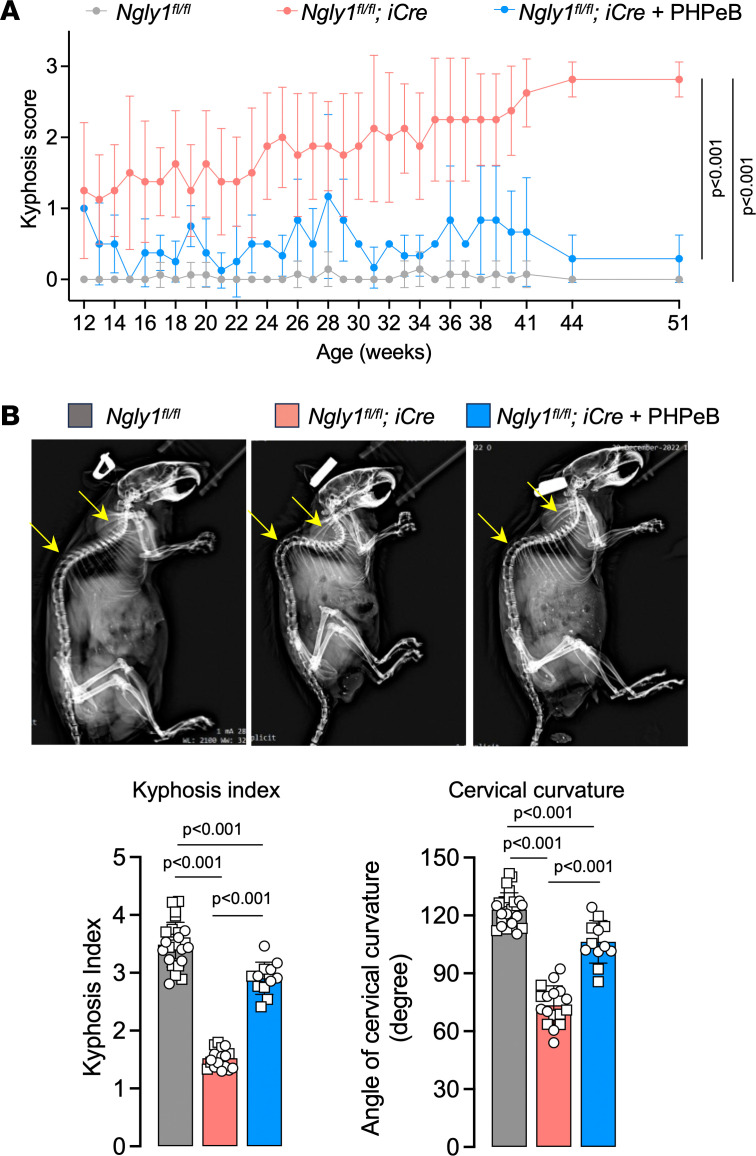
rAAV.PHPeB treatment rescues kyphosis in *iNgly1* mice. (**A**) Kyphosis scores of *Ngly1^fl/fl^* mice and *Ngly1^fl/fl^* *iCre* mice with or without rAAV.PHPeB treatment over time. *N* ≥ 12 mice per group with similar female/male ratios in each group. (**B**) Representative x-ray images showing kyphosis (yellow arrows). Quantification of kyphosis index (KI) and cervical curvature angle is shown below the images. *N* = 12 to 25 mice per group. In **A**, data are reported as the mean ± SD. Statistical analysis was performed by 2-way ANOVA, followed by Tukey’s multiple comparisons test. In **B**, data are mean ± SD of biological repeats; each white dot represents an individual mouse (circle: female; square: male). Statistical analysis was performed by 1-way ANOVA followed by Tukey’s multiple comparisons test.

To examine whether a single dose of rAAV.PHPeB treatment on P56 could afford durable therapeutic efficacy, another cohort of mice were monitored until 14 months of age, when over half of untreated *iNgly1* mice succumbed to the disease (Figure 6A). rAAV.PHPeB treatment rescued the lethality and modestly but significantly improved body weight at 14 months of age (Figure 6, A and B). Calbindin^+^ Purkinje cells and motor function were largely preserved in *iNgly1* mice receiving rAAV.PHPeB treatment (Figure 6, C–E). Kyphosis was significantly corrected and stabilized in rAAV.PHPeB-treated *iNgly1* mice when assessed until 14 months of age (Figure 7).

**Figure 8:**
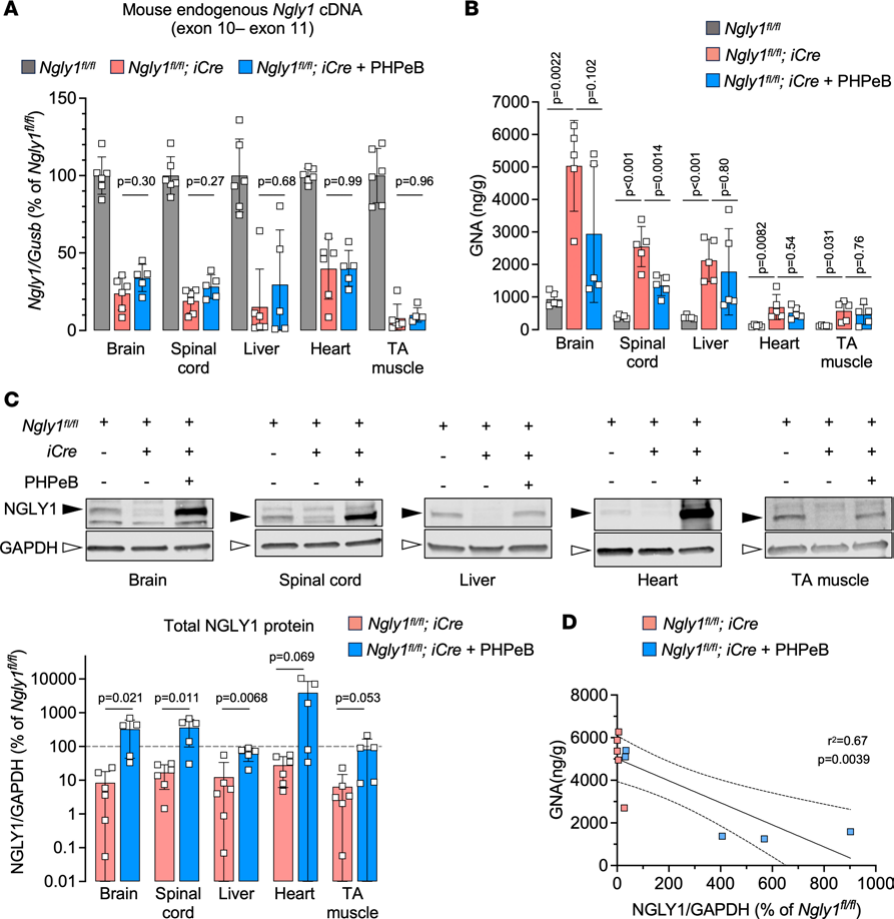
rAAV.PHPeB treatment lowers GNA in the CNS of *iNgly1* mice. (**A**) Quantification of mouse endogenous *Ngly1* mRNA expression (cDNA) in the brain, spinal cord, liver, heart, and TA muscle. *N* = 5 to 6 mice per group. (**B**) GNA levels in various tissues of *Ngly1^fl/fl^* mice and *Ngly1^fl/fl^ iCre* mice with or without rAAV.PHPeB treatment. *N* = 5 mice per group. (**C**) Representative Western blotting images of NGLY1 protein expression in the brain, spinal cord, liver, heart, and TA muscle of the mice. Quantification of NGLY1 signal (normalized to GAPDH expression) is shown below the images. *N* = 5 to 6 mice per group. (**D**) Correlation between GNA level (*y* axis) and NGLY1 protein expression (*x* axis) in the brain of *Ngly1^fl/fl^ iCre* mice with or without rAAV.PHPeB treatment. Each dot represents 1 animal. In **A**–**C**, data are mean ± SD of biological repeats; each white square represents an individual male mouse. In **A** and **B**, statistical analysis was performed by 1-way ANOVA followed by Tukey’s multiple comparisons test. In **C**, statistical analysis was performed by 2-tailed Student’s *t* test. In **D**, statistical analysis was performed by simple linear regression.

At the molecular level, we verified that endogenous *Ngly1* mRNA knockdown was comparable between the *iNgly1* mice with or without rAAV.PHPeB treatment (Figure 8A), ruling out inefficient gene knockout as a reason for phenotype difference between the 2 groups. Surprisingly, we did not observe GNA reduction in peripheral tissues, including liver, heart, and TA muscle (Figure 8B), despite NGLY1 protein restoration at levels near or above normal (Figure 8C). However, we cannot rule out the possibility that the anti-NGLY1 antibody used in Western blotting may recognize the human NGLY1 protein more efficiently than mouse NGLY1 protein. Although rAAV.PHPeB treatment significantly reduced GNA levels in the spinal cord and most *iNgly1* mouse brains, it failed to rescue elevated brain GNA levels in 2 *iNgly1* mice (Figure 8B). Western blotting using tissue lysates revealed that these 2 brain samples contained much less NGLY1 protein than the other 3 samples in the same group, likely due to technical variations leading to inefficient gene delivery to the brain (Figure 8C). Therefore, the inverse correlation between GNA levels and NGLY1 protein abundance in the brain (Figure 8D) indicates that efficient gene delivery is required to effectively reduce GNA in the brain.

## Discussion

The groundbreaking discovery that a noninvasive systemic administration of rAAV9 can cross the BBB and achieve widespread CNS gene delivery (56, 57) has opened an exciting avenue to address neurological diseases, especially the conditions afflicting broad CNS compartments. CNS-targeted IV rAAV gene therapy has been employed in several clinical applications, exemplified by onasemnogene abeparvovec (aka Zolgensma), an FDA-approved treatment for spinal muscular atrophy (SMA) (58). In postmortem tissues from 2 patients with SMA receiving Zolgensma at 1.1 × 10^14^ vg/kg, transgene biodistribution was evident throughout the CNS, reaching 0.1–1 vg/diploid genome in most brain and spinal cord samples (59). Despite the encouraging preclinical and clinical results of IV rAAV9 for SMA and other neurological diseases (60), a recent study failed to demonstrate the effectiveness of IV rAAV9 gene replacement therapy (1 × 10^14^ vg/kg) in an *Ngly1^–/–^* Sprague-Dawley rat model (43). It was likely attributable to the poor CNS biodistribution of vector DNA that was below 0.01 vg/diploid genome in most brain regions. In contrast, ICV administration resulted in therapeutic efficacy in the same *Ngly1^–/–^* rat model, which was correlated with approximately 10-fold higher vector DNA biodistribution in the CNS (43). Therefore, we hypothesized that IV administration of a more potent BBB-crossing capsid vector can lead to meaningful therapeutic efficacy for NGLY1 deficiency and have translational value.

The unrealized potential of IV rAAV gene therapy for a broad range of neurological diseases has led to considerable efforts to engineer AAV capsid for higher BBB-crossing efficiency in multiple animal species. Notably, most resulting AAV capsid variants exhibit a species-specific property, i.e., they retain enhanced performance only in the animal species that is originally used to identify them. For example, PHP.B and PHPeB capsids were shown to significantly outperform AAV9 in C57BL/6 mice (45, 46, 61). Although the superb BBB-crossing property of neither capsid translates to NHPs (62, 63), both capsids provide powerful tools to investigate the feasibility of CNS-targeted IV rAAV gene therapy for neurological diseases, such as Rett syndrome (64), fragile X syndrome (65), Dravet syndrome (66), Niemann-Pick disease (67), Pompe disease (68), and synucleinopathy (69). Recently, exciting progress has been made to engineer AAV capsid variants that can drastically outperform AAV9 in crossing the primate BBB (48–51), paving the way for future clinical development of CNS-targeted IV rAAV gene therapy. However, these primate-specific capsids do not perform equally well in mice (48) and therefore are not suitable for preclinical testing in disease mouse models.

Utilizing the PHPeB vector for NGLY1 deficiency preclinical gene therapy study faces 2 major challenges. First, *Ngly1^–/–^* mice on C57BL/6 background are embryonically lethal (26), precluding rAAV administration in postnatal animals. Second, the superb CNS-crossing property of PHPeB does not translate to rats, so the *Ngly1^–/–^* rat model is unlikely to respond. To overcome these challenges, we first generated the *iNgly1* mouse model on a C57BL/6 background that showed 80%–90% reduction in *Ngly1* expression, concomitant with disease phenotype that recapitulated human NGLY1 deficiency, including elevated GNA, failure to thrive, loss of Purkinje cells, motor impairment, and kyphosis. This mouse model allowed us to leverage the PHPeB capsid to study whether enhanced CNS penetration of IV rAAV gene therapy can be efficacious for NGLY1 deficiency and to compare with the gold-standard AAV9 capsid. As expected, PHPeB vector substantially outperformed rAAV9 for CNS gene transfer following IV administration in symptomatic *iNgly1* mice and resulted in near-complete phenotypic correction when examined at 8 weeks after injection. The PHPeB-mediated therapeutic efficacy was also evident at 14 months postinjection, supporting the durability of gene replacement therapy for NGLY1 deficiency.

Clinical translation of IV rAAV gene therapy for neurological diseases such as NGLY1 deficiency is not without challenges. The preexisting anti-AAV capsid neutralizing antibody (NAb) in the general human population can compromise or abolish gene transfer via the bloodstream. To diminish circulating NAb titers to a level permissive to rAAV administration, a pretreatment of immunoglobin-cleaving enzymes has shown promising results in mouse and NHP studies (70, 71). Another major concern is the requirement of high doses of IV rAAV to enable BBB penetration, which has been associated with toxicities in clinical settings (72) and high vector manufacturing cost. Recently, several engineered AAV capsids were identified to efficiently cross NHP (48–51) and humanized (73) BBB. These emerging capsids can potentially afford adequate pan-CNS gene transfer with lower doses and therefore improve the safety and practicality of CNS-targeted IV rAAV gene therapy.

In summary, our study reinforces the feasibility of rAAV gene replacement therapy for NGLY1 deficiency and suggests that IV delivery can lead to therapeutic efficacy provided adequate CNS penetration using a suitable capsid vector.

## Methods

### Sex as a biological variable

We included both sexes in most experiments and noted males and females as squares and circles, respectively. In the Calbindin immunohistochemistry experiments, we assessed only male mice as a representative sex, but we expected the results to be relevant to female mice as well.

### Animal use and treatment

The *Ngly1^fl/fl^ iCre* mouse line was generated by crossing a mouse line harboring *loxP*-flanked (floxed) *Ngly1* (a gift from Tadashi Suzuki, RIKEN Global Research Cluster, Wako, Saitama, Japan) and the UBC-Cre-ERT2 driver line (The Jackson Laboratory, strain: 007001) and is homozygous for the floxed *Ngly1* allele (*Ngly1^fl/fl^*) and hemizygous for the inducible *UBC-Cre-ERT2* transgene (*iCre*). The day of birth was designated P0. *Ngly1^fl/fl^ iCre* mice were genotyped by PCR using primers Cre-F: 5′-GACGTCACCCGTTCTGTTG-3′ and Cre-R: 5′-AGGCAAATTTTGGTGTACGG-3′, which generates a 475 bp PCR amplicon indicating the presence of the *iCre* transgene. Tamoxifen (MilliporeSigma, T5648) was dissolved in corn oil (MilliporeSigma, C8267) to a concentration of 2 mg/mL (for P7–P9 pups) or 20 mg/mL (for P31–P33 weanlings), then administered to mice via intraperitoneal injection at a dose of 75 mg/kg. AAV vectors were diluted in Dulbecco’s PBS (MilliporeSigma, D8537-6X500ML), and 300 μL of diluted AAV vector was injected into mice via tail vein at a dose of 1.5 × 10^12^ vg/mouse. The number of animals used in each assay is detailed in the Supporting Data Values file. We included a larger cohort of animals for behavioral assays because of the inherent high variability associated with those assays and randomly selected a sex-balanced subset of animals for molecular analyses (e.g., GNA, DNA, RNA, protein quantification) to lower the cost. All data from the assessed animals are reported in the Supporting Data Values file. The body weight measurement was a combination of cross-sectional and longitudinal studies, resulting in some animals not being assessed at all time points.

#### Western blotting.

Mouse tissues were homogenized using TissueLyser II (QIAGEN) in ice-cold T-PER (Thermo Fisher Scientific, 78510) with protease inhibitor (Roche, 4693159001). Total protein concentration was determined by Pierce BCA Protein Assay Kit (Thermo Fisher Scientific, 23225). Protein lysates normalized for total protein amount were boiled with 4× Laemmli sample buffer (Bio-Rad, 1610747) at 99°C for 5 minutes. Primary antibodies were rabbit anti-NGLY1 (MilliporeSigma, HPA036825, 1:1,000) and mouse anti-GAPDH (Abcam, ab8245, 1:10,000). Secondary antibodies were LI-COR IRDye 680RD goat anti-mouse IgG (H + L) (LI-COR Biosciences, 926-68070, 1:7,000) and LI-COR IRDye 800CW goat anti-rabbit IgG (H + L) (LI-COR Biosciences, 926-32211, 1:7,000). Blot membranes were imaged by LI-COR scanner (Odyssey) and quantified by LI-COR software.

### Immunohistochemistry

Mice were perfused intracardially with ice-cold PBS, and tissues were dissected and immersion-fixed in 4% paraformaldehyde (Electron Microscopy Sciences, 15710) for 16–24 hours at 4°C followed by embedding in paraffin. Sectioning and immunohistochemistry with rabbit anti-Calbindin antibody (Abcam, ab229915, 1:3,000) were performed by the Morphology Core of University of Massachusetts Chan Medical School. Slides were imaged with a Leica Thunder Microscope. Quantification of the Calbindin^+^ cell number was performed using ImageJ Fiji. Three to 4 sagittal sections from a brain hemisphere were analyzed for each mouse as previously described (74). The multi-point tool in ImageJ was used to mark the number of Calbindin^+^ cells; the length of the Purkinje cell layer was measured with the segmented line tool.

### X-ray radiography and kyphosis assessment

Radiographs of anesthetized mice in lateral position were obtained using an x-ray chamber (Trident digital x-ray machine, Hologic) at the Bone Core of University of Massachusetts Chan Medical School as previously reported (75). Quantification of kyphosis index was performed as previously described (75, 76). Briefly, a line AB was drawn from the posterior edge of the seventh segment of cervical vertebra (C7) to the posterior edge of the sixth lumbar spine (L6); another line CD was from the dorsal border of the vertebral body farthest from and perpendicular to the line AB. The lengths of AB and CD were measured using ImageJ Fiji; kyphosis index was defined as the ratio AB/CD.

### Kyphosis score

Kyphosis severity was scored as previously described (77). Briefly, each mouse is observed on a flat surface. If the mouse can easily straighten its spine when it walks and does not have persistent kyphosis, it is scored 0. If the mouse has mild kyphosis but can straighten its spine, it is scored 1. If the mouse cannot straighten its spine completely and exhibits persistent but mild kyphosis, it is scored 2. If the mouse maintains pronounced kyphosis while it walks or rests, it is scored 3.

### Behavioral tests

All behavioral tests were performed at the Mouse Behavioral Core Facility of University of Massachusetts Chan Medical School. Mice were maintained under a 12-hour light/12-hour dark cycle, and all behavioral tests were performed during the light phase. Mice were acclimated to the test room environment for 30 minutes before testing. Unless otherwise stated, all equipment was cleaned with 70% ethanol between trials to provide a standardized testing environment.

#### Rotarod test.

Each mouse underwent 2 days of training sessions and 1 day of test sessions. Each session consisted of 3 separate 4-minute trials with a 15-minute interval between trials. The rotarod’s speed was accelerated from 4 to 40 rpm in 4 minutes at an accelerating rate of 0.15 revolutions per second. The time that each animal stayed on the rolling rod without falling was recorded. Each mouse was subjected to 3 tests, and the best performance was recorded. If a mouse remained on the rod at the end of the 4-minute trial, a time of 240 seconds was recorded.

#### CatWalk gait analysis.

Gait analysis was performed using the CatWalk XT system (Noldus) (78). Mice were trained on an enclosed glass platform at least 3 times before gait analysis began. Each mouse was placed on the open end of the glass plate and allowed to walk across the entire glass floor voluntarily. A high-speed camera under the platform captured the image of each footprint and transmitted the recorded data to the gait analysis software (CatWalk XT, version 10.6; Noldus). Max intensity is the maximum intensity of a paw at maximum contact to the glass platform, ranging from 0 to 255. Single stance is the duration of ground contact by a single paw. Stride length is the distance between successive placements of the same paw.

### AAV vectors

The therapeutic AAV vector construct contains codon-optimized human *NGLY1* cDNA driven by the CMV/CB promoter. This cassette was packaged into a single-stranded AAV9 and AAV.PHPeB vectors. AAV vectors were produced using standard triple-transfection method and purified by CsCl or iodixanol ultracentrifugation. rAAV titers were determined by droplet digital PCR (for rAAV genome) and gel electrophoresis followed by silver staining (for rAAV capsid).

### Quantification of vector genome copies and cDNA by droplet digital PCR

DNA and RNA were extracted from mouse tissues using the AllPrep DNA/RNA Mini Kit (QIAGEN, 80204). RNA was reverse-transcribed using the High-Capacity cDNA Reverse Transcription kit (Thermo Fisher Scientific, 43-688-13). The endogenous mouse *Ngly1* cDNA was quantified in a multiplexed reaction using TaqMan reagents targeting mouse *Ngly1* exons 10–11 (Thermo Fisher Scientific, assay ID: Mm01319643_m1) and *Gusb* (Thermo Fisher Scientific, assay ID: Mm01197698_m1). The vector genome copy number was determined in a multiplexed reaction using TaqMan reagents targeting *opt-hNGLY1* (Thermo Fisher Scientific, assay ID: AP329CT) and *Tfrc* (Thermo Fisher Scientific, 4458367). The *opt-hNGLY1* cDNA was quantified in a multiplexed reaction using TaqMan reagents targeting *opt-hNGLY1* (Thermo Fisher Scientific, assay ID: AP329CT) and *Gusb* (Thermo Fisher Scientific, assay ID: Mm01197698_m1).

### Quantitation of GNA

GNA in sera and tissues was quantified by Oakland Analytics as previously described (24, 43). Briefly, samples were homogenized in water by bead milling and mixed with 4× volumes of ice-cold internal standard solution (9:1 acetonitrile/water containing 0.05% formic acid and 50 ng/mL GNA-d3). The mixture was centrifuged at 6,100*g* for 30 minutes, and an aliquot of each supernatant was transferred to an autosampler plate and sealed with a cap-mat. The sample was separated by high-pressure liquid chromatography (Shimadzu VP Series 10 System) and subsequently analyzed using tandem mass spectrometry (Applied Biosystems/MDS SCIEX API 4000).

### Statistics

Data were presented as mean ± SD. Comparisons between 2 groups were analyzed by *t* test (2 sided). Any comparison among multiple groups was analyzed by 1-way or 2-way ANOVA followed by pairwise comparison, corrected for multiple comparisons. All statistical tests were performed with GraphPad Prism 10.

### Study approval

All animal experiments were reviewed and approved by the Institutional Animal Care and Use Committee of the University of Massachusetts Chan Medical School.

### Data availability

Data are available in the Supporting Data Values XLS file.

## Author contributions

AD, GG, and DW designed the study. AD, KY, XZ, CZ, JL, LR, and NL conducted experiments. KY and NY provided the *Ngly1^fl/fl^* *iCre* mouse line. AD, GG, NY, and DW analyzed the data. AD wrote the original draft. AD, GG, and DW reviewed and edited the manuscript.

## Supplementary Material

Unedited blot and gel images

Supporting data values

## Version 1 08/13/2024

In-Press Preview

## Version 2 10/08/2024

Electronic publication
