# Supplementary material for: Systemic gene therapy corrects the neurological phenotype in a mouse model of NGLY1 deficiency
Source: JCI Insight. 2024 Oct 8;9(19):e183189. doi: 10.1172/jci.insight.183189 (PMC11466192; doi:10.1172/jci.insight.183189)
Supplement: Unedited blot and gel images [file jciinsight-9-183189-s009.pdf]

Raw data Figure 1. Ngly1 inducible KO C57BL/6 (B6) mice as an animal model of NGLY1 deficiency

Full unedited gel for Figure 1E

(Representative blot)

Brain

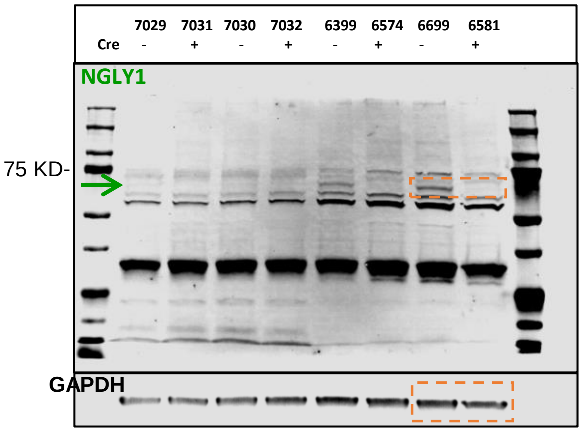

Spinal cord

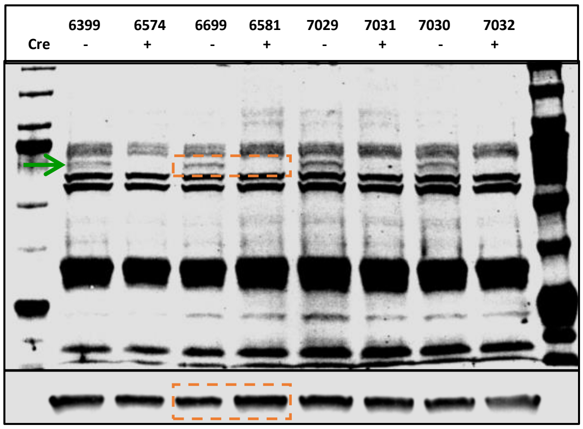

Liver

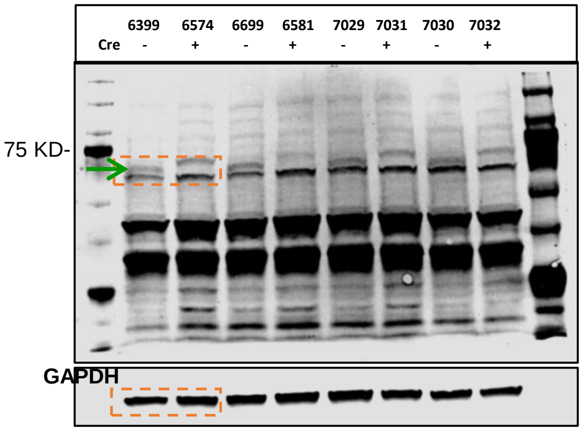

Heart

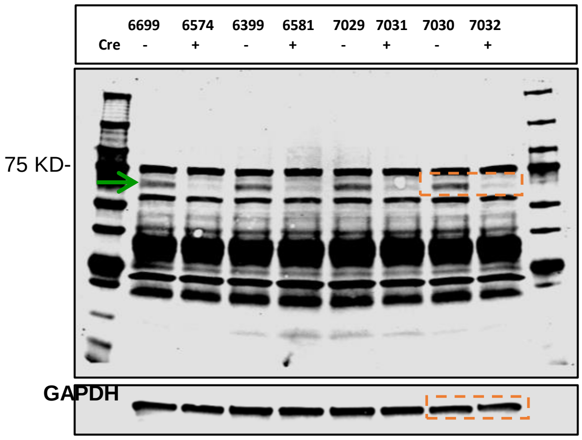

TA

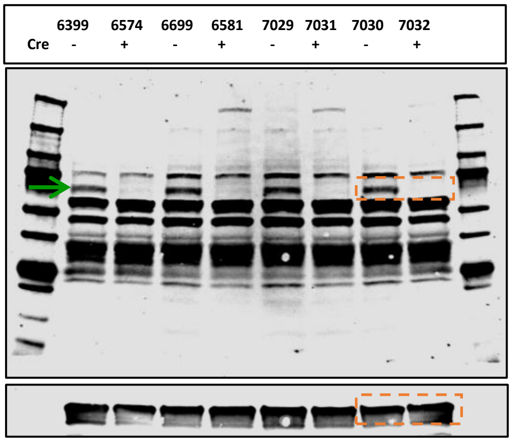

1st Ab: anti Rabbit NGLY-1 Antibody (Sigma-Aldrich, HPA036825) 1:1000; anti GAPDH 1:10000

2nd Ab: Goat anti Rabbit 800; Goat anti Mouse 680 Gel: 10% （20ug protein per sample）

Raw data Figure 1. Ngly1 inducible KO C57BL/6 (B6) mice as an animal model of NGLY1 deficiency

Full unedited gel for Figure 1E

(n=4 mice/group for quantification)

Brain

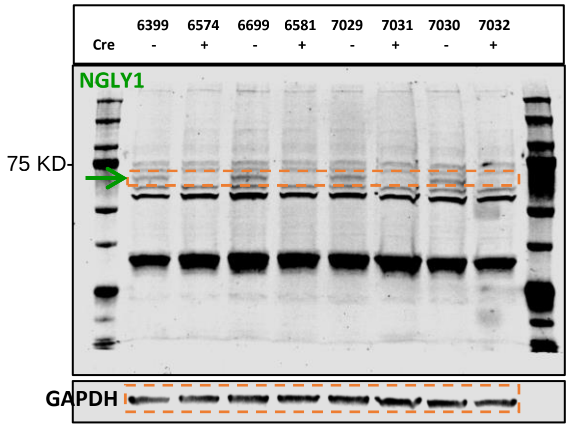

Spinal cord

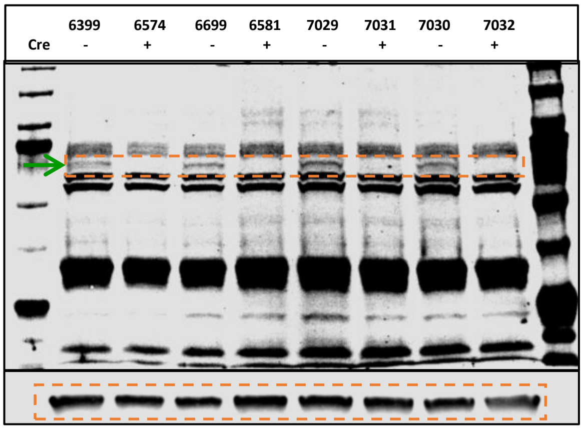

Liver

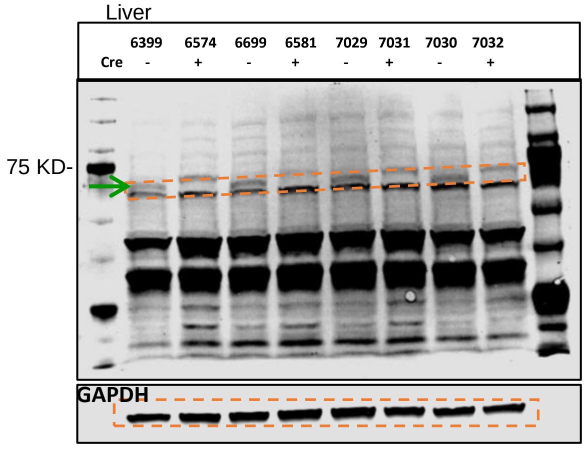

Heart

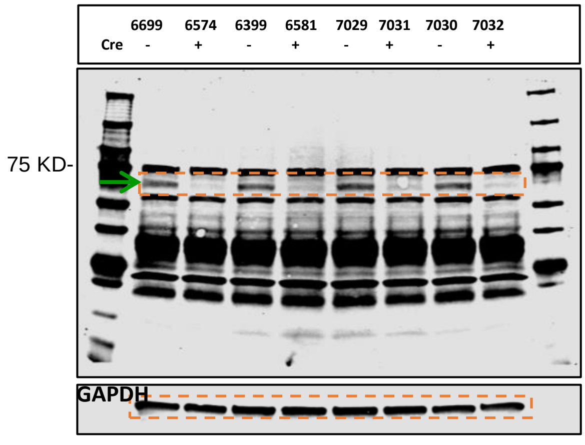

TA

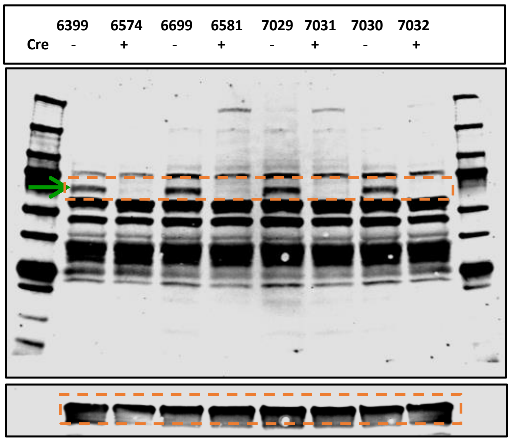

1st Ab: anti Rabbit NGLY-1 Antibody (Sigma-Aldrich, HPA036825) 1:1000; anti GAPDH 1:10000

2nd Ab: Goat anti Rabbit 800; Goat anti Mouse 680 Gel: 4-20% （20ug protein per sample）

Raw data Figure 3. Comparing rAAV9 and rAAV.PHPeB for gene delivery and expression efficiency

Full unedited gel for Figure 3E

(Representative blot)

Brain

**Male**	**7385**	**7861**	**7724**	**7665**	**7864**	**7863**	**M**
**Cre**	**-**	**+**	**+**	**+**	**-**	**+**	
**rAAV**	**-**	**PBS**	**AAV9**	**PHPeB**	**-**	**PHPeB**	

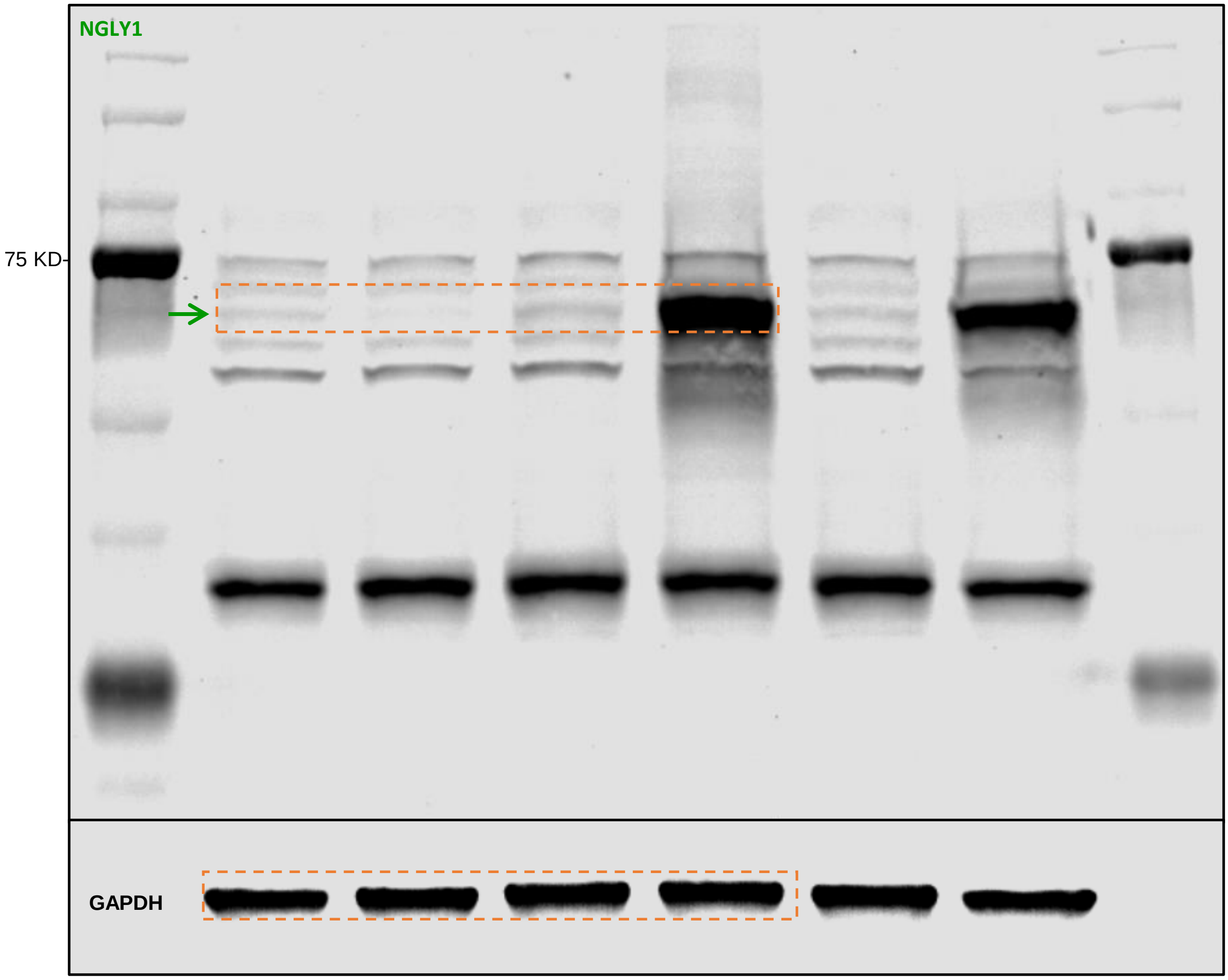

1st Ab: anti Rabbit NGLY-1 Antibody (Sigma-Aldrich, HPA036825) 1:1000; anti GAPDH 1:10000

2nd Ab: Goat anti Rabbit 800; Goat anti Mouse 680 Gel: 10% （20ug protein per sample）

Raw data Figure 3. Comparing rAAV9 and rAAVPHPeB for short-term therapeutic efficacy

Full unedited gel for Figure 3E

(n=5-8 mice/group for quantification)

Brain

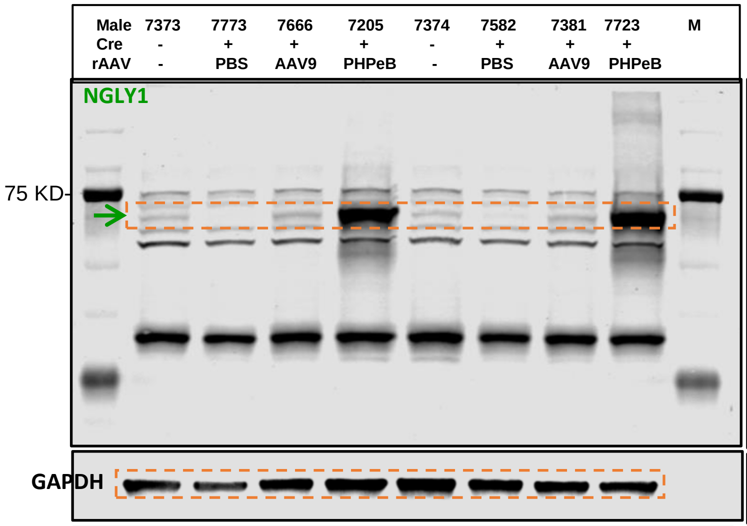

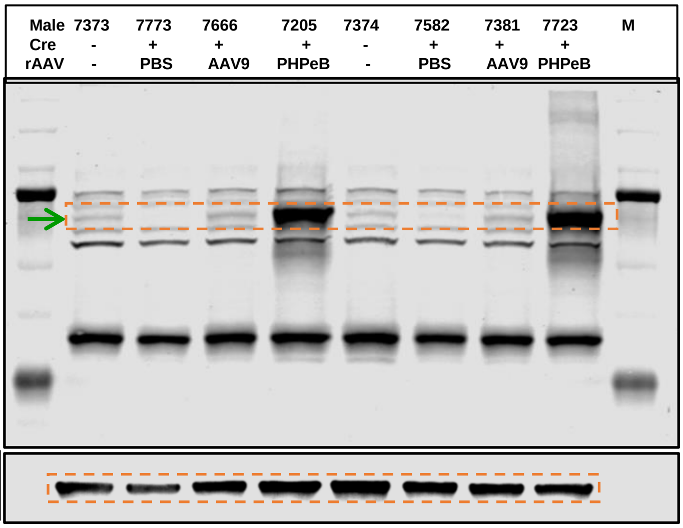

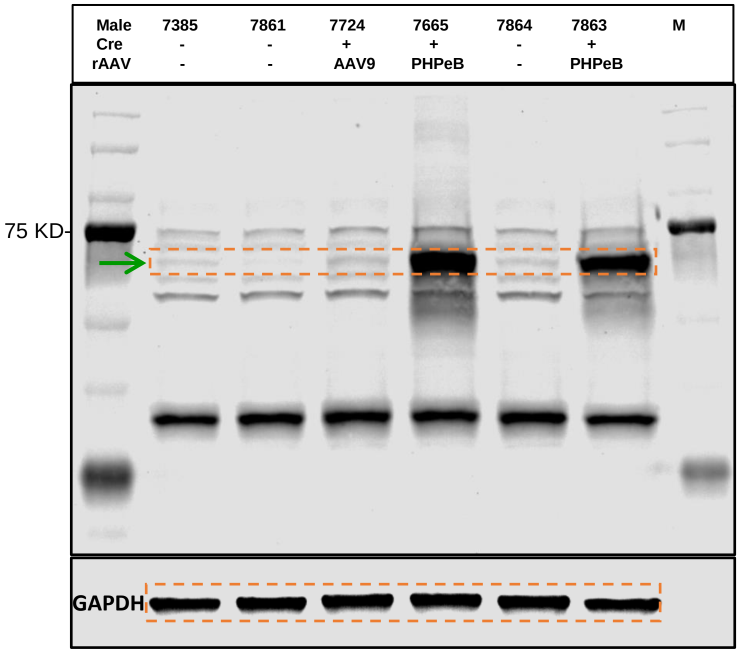

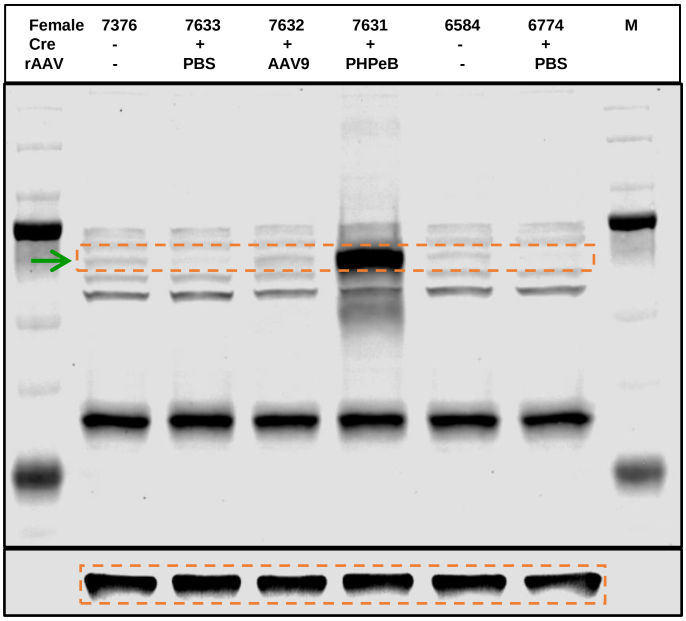

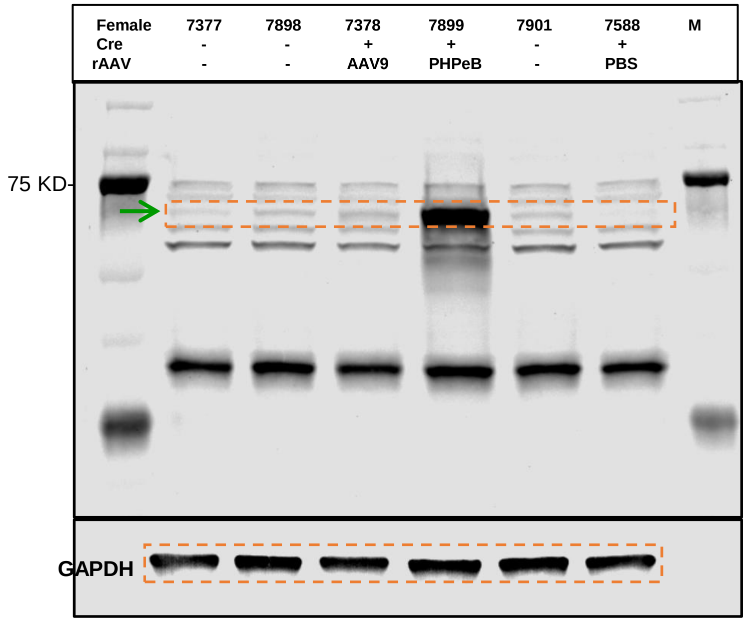

1st Ab: anti Rabbit NGLY-1 Antibody (Sigma-Aldrich, HPA036825) 1:1000; anti GAPDH 1:10000

2nd Ab: Goat anti Rabbit 800; Goat anti Mouse 680 Gel: 10% （20ug protein per sample）

Raw data Figure 8. rAAV.PHPeB treatment lowered GNA in the CNS of iNgly1 mice.

Full unedited gel for Figure 8C

(Representative blot)

Brain

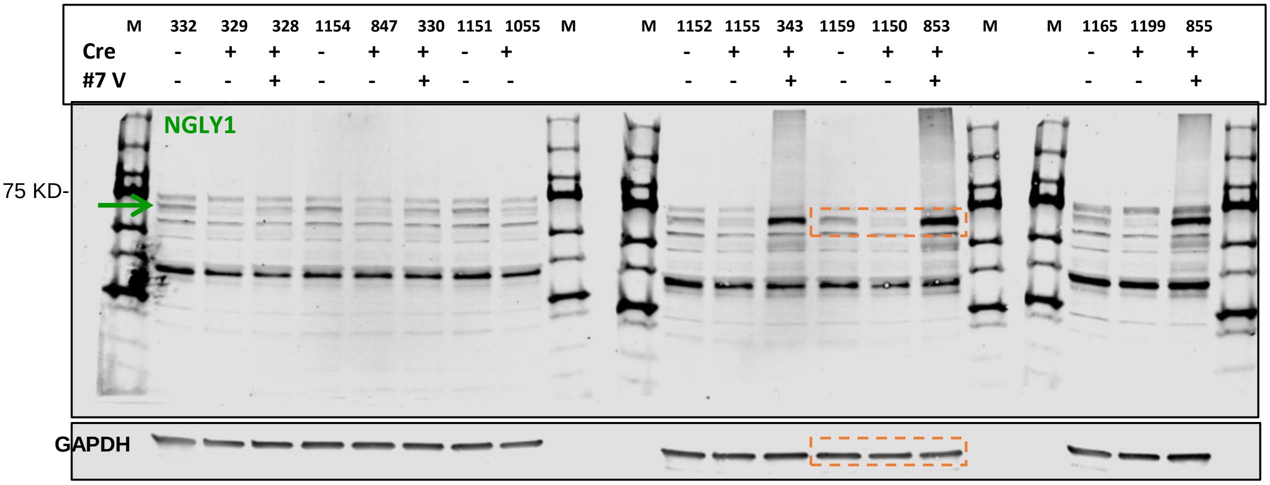

Spinal cord

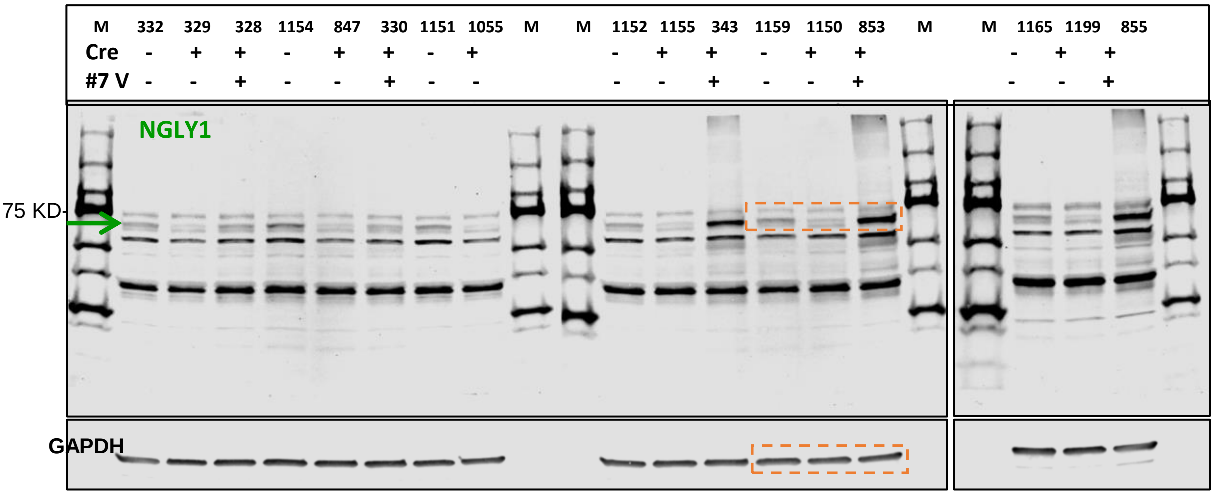

Liver

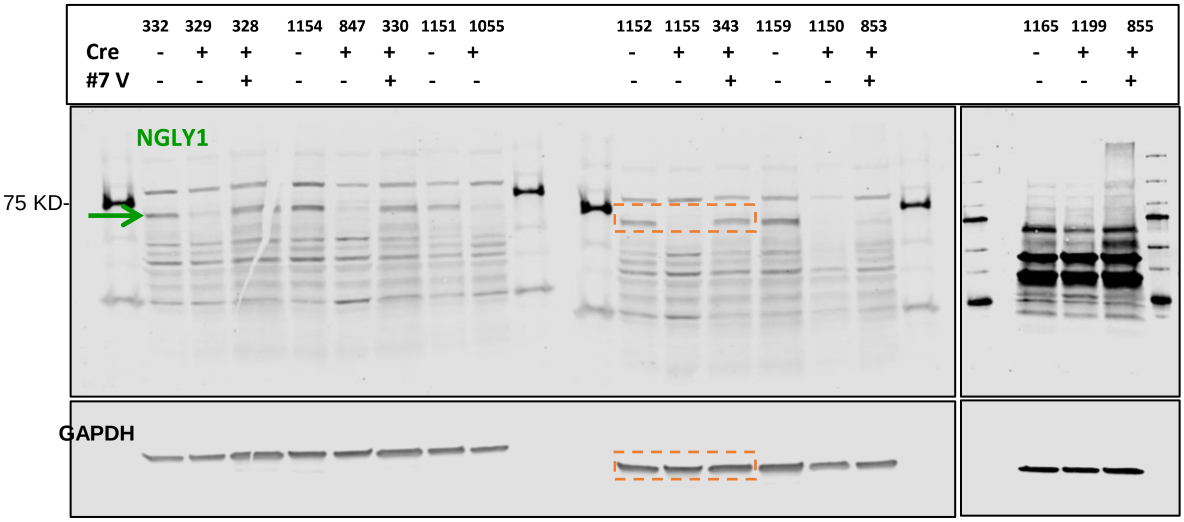

1st Ab: anti Rabbit NGLY-1 Antibody (Sigma-Aldrich, HPA036825) 1:1000; anti GAPDH 1:7500

2nd Ab: Goat anti Rabbit 800; Goat anti Mouse 680 Gel: 4-20% (20ug protein per sample)

#7V: rAAV PHPeB vector

Raw data Figure 8. rAAV.PHPeB treatment lowered GNA in the CNS of iNgly1 mice.

Full unedited gel for Figure 8C

(Representative blot)

Heart

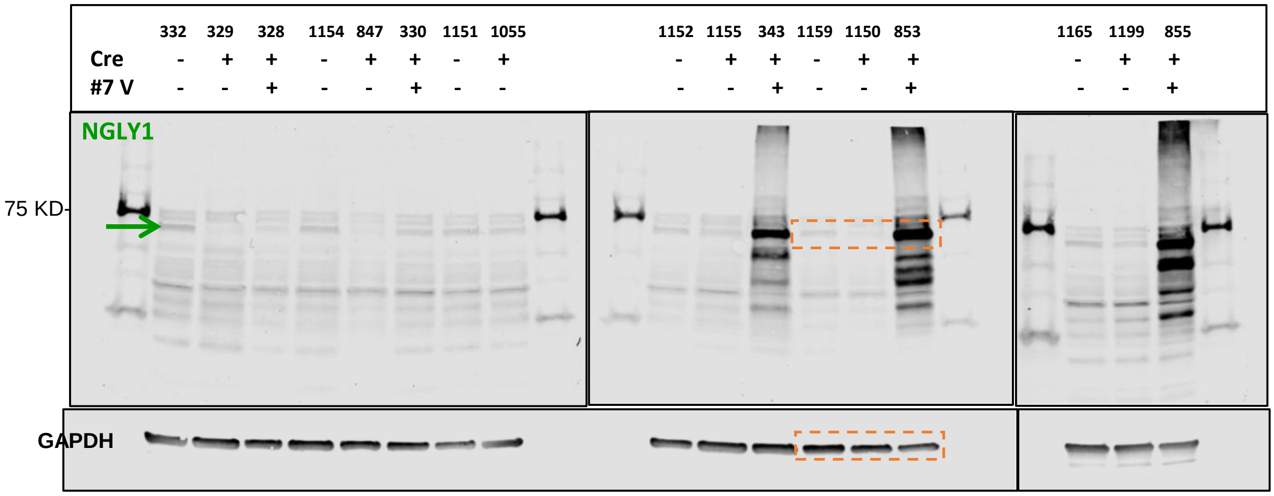

TA

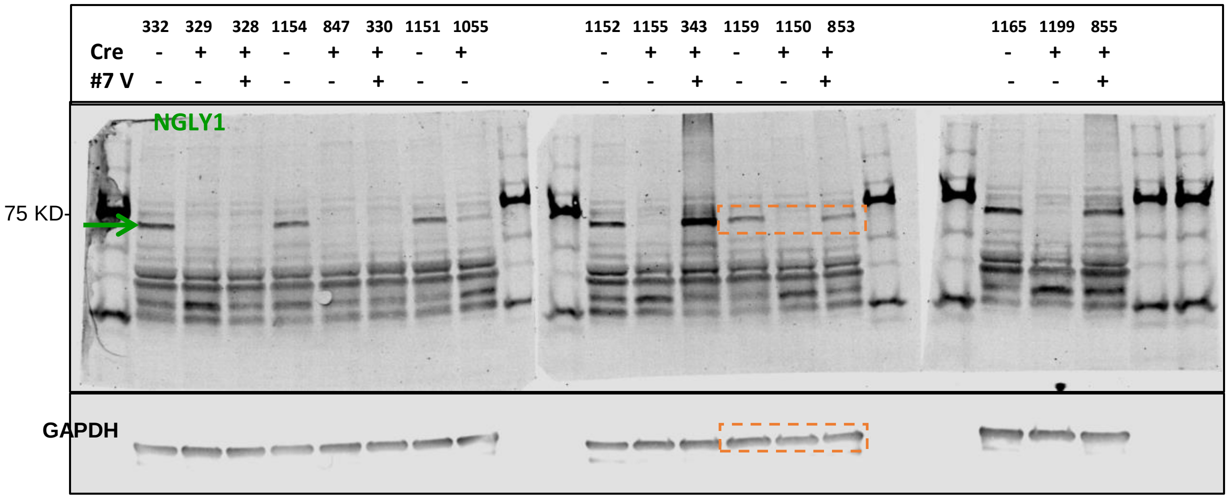

1st Ab: anti Rabbit NGLY-1 Antibody (Sigma-Aldrich, HPA036825) 1:1000; anti GAPDH 1:7500

2nd Ab: Goat anti Rabbit 800; Goat anti Mouse 680 Gel: 4-20% （20ug protein per sample）

#7V: rAAV PHPeB vector

Raw data Figure 8. rAAV.PHPeB treatment lowered GNA in the CNS of iNgly1 mice.

Full unedited gel for Figure 8C

(n=5 to 6 mice/group for quantification)

Brain

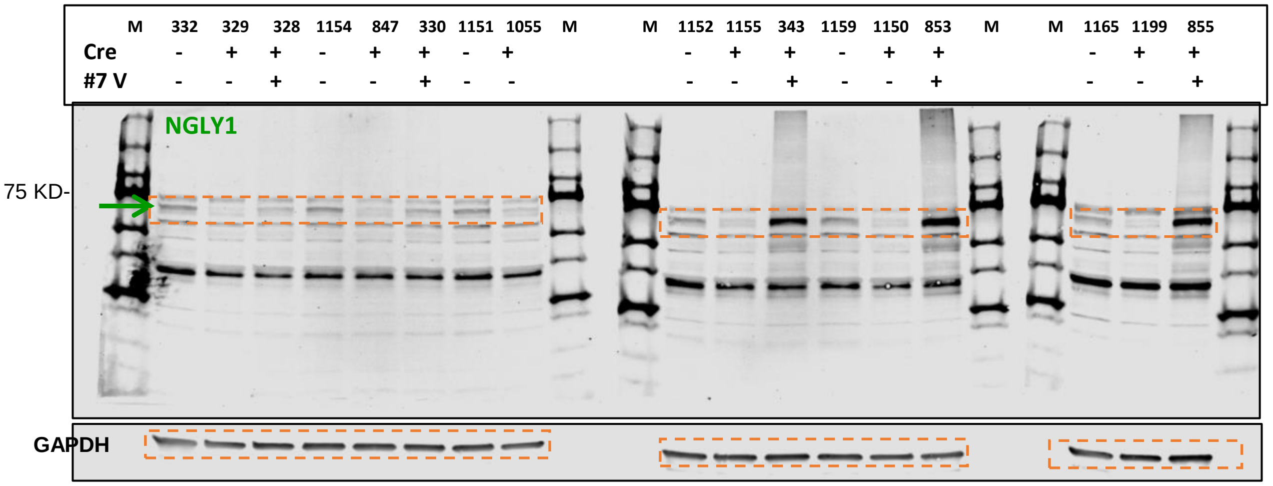

Spinal cord

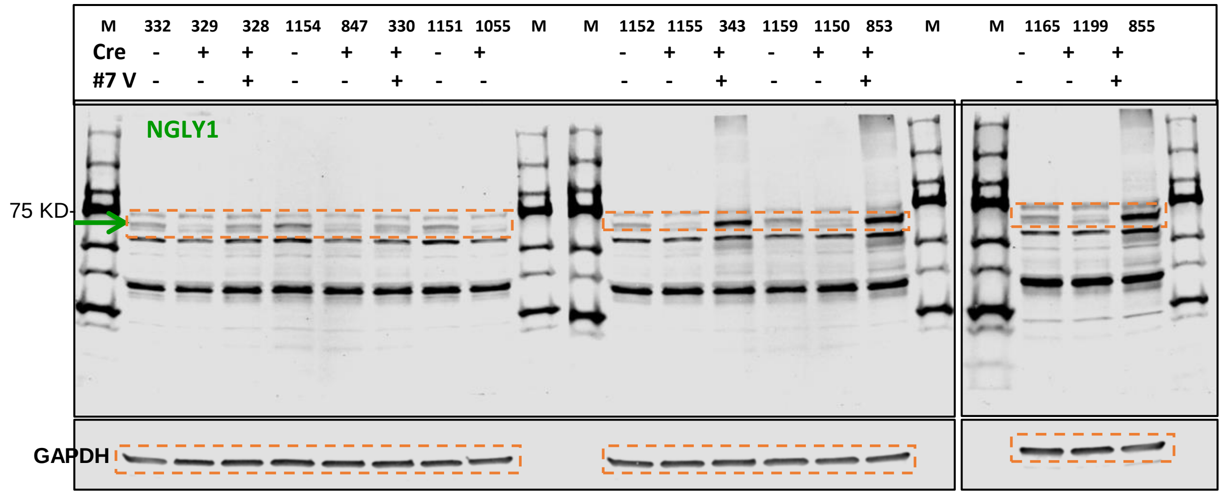

Liver

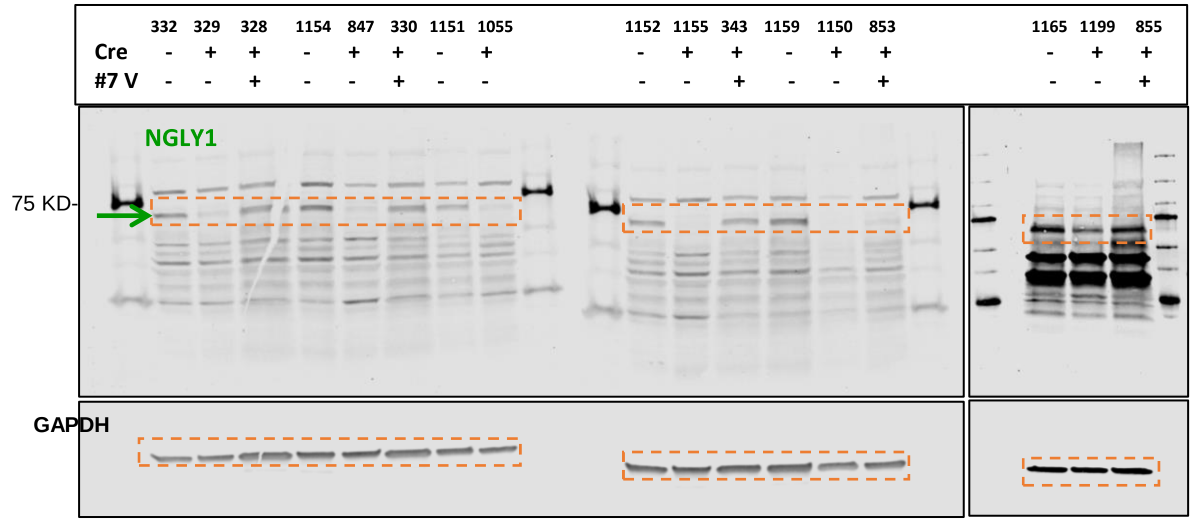

1st Ab: anti Rabbit NGLY-1 Antibody (Sigma-Aldrich, HPA036825) 1:1000; anti GAPDH 1:7500

2nd Ab: Goat anti Rabbit 800; Goat anti Mouse 680 Gel: 4-20% （20ug protein per sample）

#7V: rAAV PHPeB vector

Raw data Figure 8. rAAV.PHPeB treatment lowered GNA in the CNS of iNgly1 mice.

Full unedited gel for Figure 8C

(n=5 to 6 mice/group for quantification)

Heart

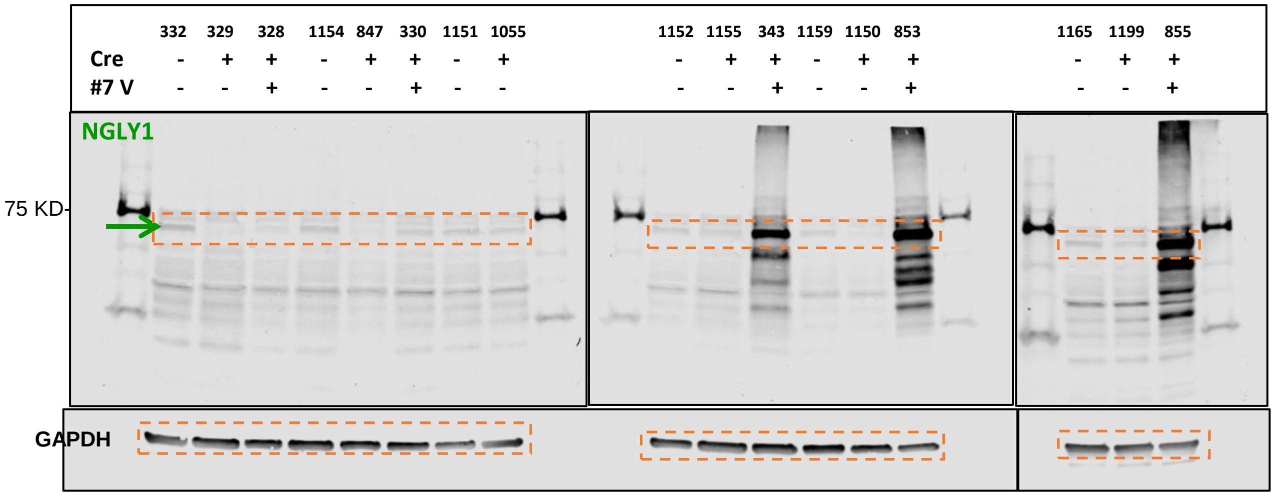

TA

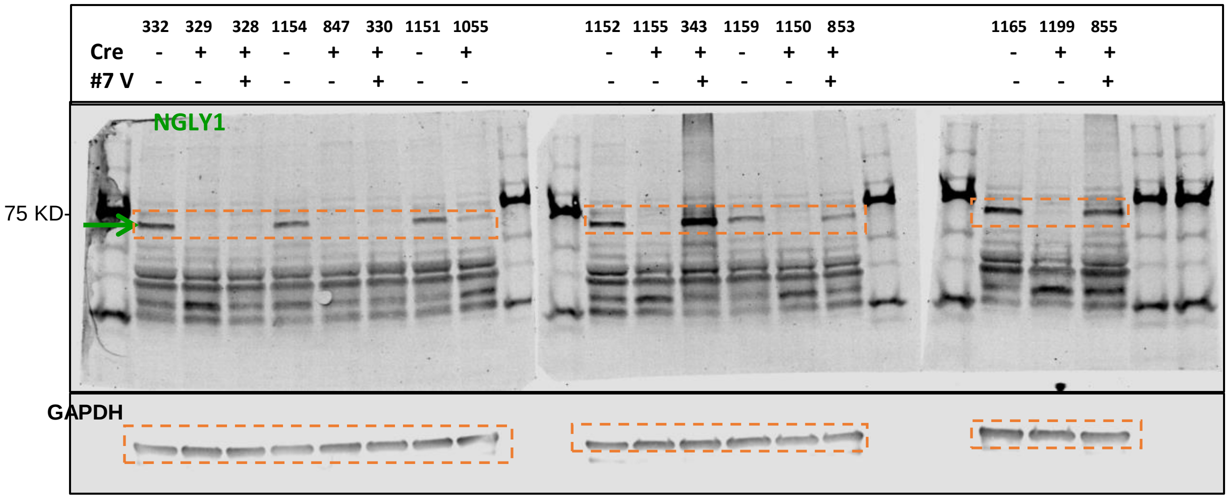

1st Ab: anti Rabbit NGLY-1 Antibody (Sigma-Aldrich, HPA036825) 1:1000; anti GAPDH 1:7500

2nd Ab: Goat anti Rabbit 800; Goat anti Mouse 680 Gel: 4-20% （20ug protein per sample）

#7V: rAAV PHPeB vector
